# Nephrogenic Systemic Fibrosis in Denmark– A Nationwide Investigation

**DOI:** 10.1371/journal.pone.0082037

**Published:** 2013-12-09

**Authors:** Tina R. Elmholdt, Anne B. B. Olesen, Bettina Jørgensen, Stinne Kvist, Lone Skov, Henrik S. Thomsen, Peter Marckmann, Michael Pedersen

**Affiliations:** 1 Institute of Clinical Medicine, Aarhus University Hospital, Aarhus University, Aarhus, Denmark; 2 Department of Dermatology, Aarhus University Hospital, Aarhus, Denmark; 3 Department of Nephrology, Aarhus University Hospital, Aarhus, Denmark; 4 Department of Dermato-Allergology, Copenhagen University Hospital, Gentofte, Denmark; 5 Department of Diagnostic Radiology, Copenhagen University Hospital, Herlev, Denmark; 6 Department of Internal Medicine, Copenhagen University Hospital, Roskilde, Denmark; 7 Department of Nephrology, Odense University Hospital, Odense, Denmark; 8 MR Research Centre, Aarhus University, Aarhus, Denmark; Institute of Automation, Chinese Academy of Sciences, China

## Abstract

**Background:**

Nephrogenic systemic fibrosis is a debilitating and painful disorder with an increased stimulation of the connective tissue in the skin and systemic tissues. The disease is associated with exposure to gadolinium-based contrast agent used in magnetic resonance imaging in patients with renal impairment.

**Methods:**

The prevalence of nephrogenic systemic fibrosis has so far never been determined at a national level. In 2009, Denmark was the first country to design a guideline for the tracing of nephrogenic systemic fibrosis patients. The aim of this paper is to communicate the main findings of this quest.

**Results:**

The outcome of the nationwide investigation revealed that Denmark had 65 patients with nephrogenic systemic fibrosis and thereby the highest prevalence of nephrogenic systemic fibrosis worldwide with 65 per 5.6 million inhabitants, or 12 per million.

**Conclusions:**

The nationwide investigation in Denmark revealed the highest prevalence of NSF worldwide. This may be rooted in a high level of awareness of NSF both among doctors, politicians and, not least, the media, combined with the fact that a nationwide NSF investigation was initiated.

## Background

Nephrogenic systemic fibrosis (NSF) occurs in some patients with renal impairment following exposure to gadolinium-based contrast agent (GBCA); this serious adverse event was first identified in 1997 [1] and later published in 2000 [2]. This disease was first named nephrogenic fibrosing dermopathy [3], but was later renamed NSF due to its systemic involvement [4]–[8].

### MRI and GBCA practice in Denmark before 2006

Magnetic resonance imaging (MRI) was introduced in Denmark in 1985 and the first MRI contrast agent Magnevist® was approved by the Danish Medical Agency (DMA) in 1989. Around 1996 many believed that gadolinium-based contrast agents (GBCAs) were non-nephrotoxic and it was recommended to switch from enhanced computer tomography (CT) to enhanced MRI in patients suffering from renal dysfunction, particularly for angiographic evaluation. No evidence has been produced to the effect that Danish radiologists should use GBCAs differently from radiologists in the rest of the Western world, where doses traditionally lie in the range 0.1–0.3 mmol/kg [9].

### Course of events leading up to the nationwide investigation in Denmark

In March 2006, 20 patients suspected of having NSF were reported to the DMA from the Copenhagen University Hospital, Herlev. In August 2006, the same institution published 13 biopsy-confirmed NSF cases [10]. Due to the growing number of reports of NSF cases and the hypothesis that GBCA might serve as a potential trigger of NSF [11], the European Medicines Agency (EMA) in February 2007 recommended caution or avoidance of Omniscan® in patients with severe renal failure, and the EMA issued a general warning regarding the other GBCAs.

In Denmark, the issue was intensively covered by the media, and the Ministry of the Interior and Health (MIH) therefore requested all hospitals in Denmark to notify instances of NSF to the DMA. In September 2008, a memorandum was published by the DMA stating that the number of NSF patients in Denmark was around 30; primarily based on the cases reported from Herlev Hospital. In 2009, new cases of NSF were reported from the Aarhus University Hospital, Skejby. This called for a more methodical search. Thus, the Danish Society of Nephrology took the initiative to create a guideline for a nationwide investigation of the prevalence of NSF in Denmark. The initiative specifically targeted patients with renal impairment exposed to GBCAs from 1997 to 2010. The investigatory guideline was distributed to all Danish nephrology departments [12], and each department had to report their findings to the DMA.

This paper describes the creation and outcomes of the first nationwide investigation of NSF worldwide and it emphasises the pivotal importance of clinical awareness and active systematic tracing.

## Methods

### The guideline created for the nationwide investigation

#### Ethical statement

This study did not need approval by the institutional board as the data already was part of a public report and anonymous. The committee established to create the guideline consisted primarily of specialists from nephrological or dermatological departments. The guideline was based on national and international NSF experiences and listed the clinical symptoms and signs of NSF [13]. The guideline was drawn up to trace diagnoses of chronic, acute or terminal kidney insufficiency in which MRI contrast agents had been used. Only living patients were included because a clinical examination was essential to diagnose NSF. However, those NSF patients who had already been identified but who had died before the investigation started were included in the final report. The guideline recommended that each site of investigation appointed a clinical coordinator.

### The NSF diagnosis and challenges

**Figure 1:**
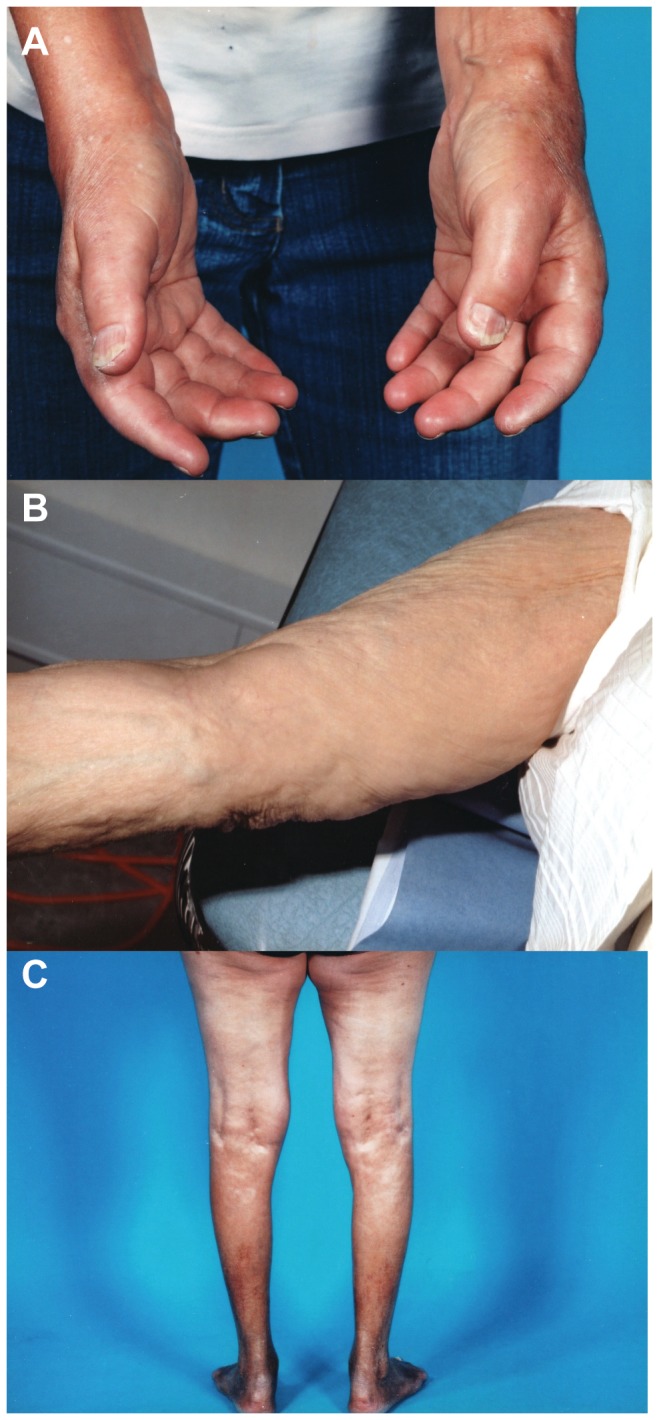
Clinical photos from nephrogenic systemic fibrosis patients. A: Tightness and hardness of the hands combined with joint contractures. B: Firm nodules establishing a cobblestone configuration. C: Tight and firm skin on lower legs.

In case of suspected NSF, the patient was referred to a doctor experienced in NSF who undertook a clinical examination [14]. The clinical examination included a thorough evaluation of the skin (Figure 1). Some patients were also assessed with a modified Rodnan skin score [15] in order to grade the severity of the fibrosis, and blood tests were drawn to exclude diseases mimicking NSF. Deep punch biopsies were taken whenever clinical suspicion of NSF was raised. The patient was diagnosed with NSF if clinical and pathological observations were consistent with the established diagnostic criteria [16] [17]. Notably, the diagnosis was challenged in some patients because they presented with late phase NSF [18].

### Data from the nationwide investigation

The MIH and the DMA collected all the data from the nationwide investigation in a report which was published in September 2010 [19]. These data included: name of hospital, GBCA(s), dosage(s), renal diagnosis, date of exposure and NSF diagnosis for each patient. Unfortunately, not all information was accessible in every case. Lack of data was mostly due to incomplete chart registering. We also asked the DMA for data regarding age, sex, status of dialysis before the triggering GBCA exposure, renal transplantation, alive or dead, the overall number of NSF patients registered in Denmark and Europe, and the overall purchase of GBCAs in Denmark in the period 1998–2011.

## Results

### Characteristics of NSF patients identified

A total of 65 patients were verified with a diagnosis of NSF [20]; they were reported from 11 different hospitals. Thirty-seven were (57%) men and 28 (43%) women (Table 1). The majority of these cases originated from Herlev and Skejby Hospital (n = 49). Their dialysis status before the triggering GBCA exposure and their renal diagnoses are described in Table 1.The first NSF patient was identified in 2003 and the latest in 2010. Five of the NSF patients underwent kidney transplantation after the NSF diagnosis had been established with the following results: two of these patients experienced improvement of their NSF symptoms [21], whereas one patient only experienced some improvement and two experienced no improvement. Three NSF patients were identified after the nationwide investigation was completed.

**Table 1:** Characteristics of the 65 NSF patients.

Sex and age		Renal diagnosis of the NSF patients	
Male/female ratio	1.3	End stage renal disease	24
Age, mean ± SD (range) years, male	58±12 (30–78)	Focal segmental glomerulosclerosis	1
Age, mean ± SD (range) years, woman	52±13 (22–81)	Epimembranous glomerulonephritis	1
		Hypertensive nephropathy	7
**Hospitals**		IgA nephritis	2
Herlev Hospital	31/65 (48%)	Chronic glomerulonephritis	4
Skejby Hospital	13/65 (20%)	Cirrhosis of the kidney	4
Aalborg Hospital	8/65(12.5%)	Haemolytic-uremic syndrome	2
Odense Hospital	3/65 (4.5%)	Polycystic kidney disease	2
Aarhus and Skejby Hospital	3/65 (4.5%)	Chronic kidney disease	3
Glostrup and Herlev Hospital	2/65 (3%)	Acute tubulointerstitial nephritis	3
Svendborg and Odense Hospital	1/65 (1.5%)	Nephropatia diabetic	5
Gentofte and Herlev Hospital	1/65 (1.5%)	Chronic Pyelonephritis	2
Rigshospitalet Hospital	1/65 (1.5%)	Lupus nephritis	1
Holstebro Hospital	1/65 (1.5%)	Renal cell carcinoma bilateral	1
Hillerød Hospital	1/65 (1.5%)	Unknown	3
		**Dialysis status prior to NSF debut**	
		Dialysis	44/65 (67.7%)
		No dialysis	16/65 (24.6%)
		Not recorded	5/65 (7.7%)

### Description of the NSF patients' GBCA exposure

**Figure 2:**
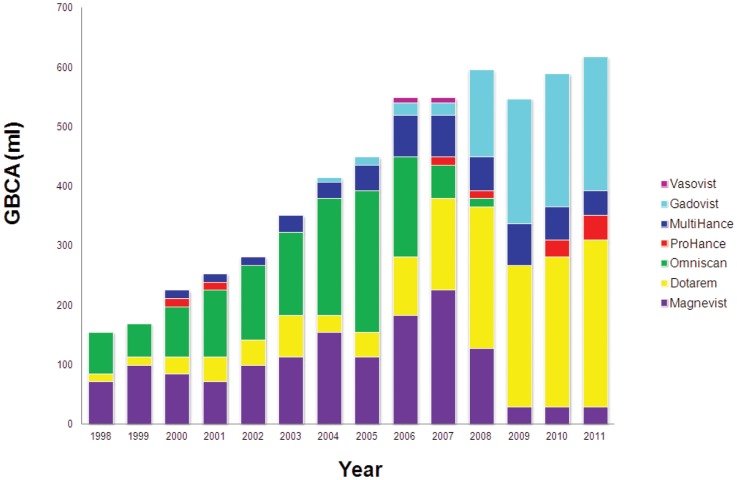
The total amount of GBCAs purchased in Denmark in the period 1998–2011. The figure illustrates a deceleration and stagnation of GBCA consumption from 2006 and onwards. Omniscan® disappeared after 2008 and sales of Magnevist® experienced a drastic decline. Instead, Dotarem® and Gadovist® increased their marked share from 2008. Magnevist®, Optimark® and Vasovist/Ablavar® are no longer marketed in Denmark, and Primovist/Eovist® is not registrered in Denmark, leaving five of nine world-wide known agents on today's market.

The 65 NSF patients were exposed to: Omniscan®, Dotarem®, Magnevist®, Multihance® and Gadovist® in a total of 110 MRI examinations (average 1.7 MRI per patient) with a mean dose of 31.5 ml GBCA. The highest cumulative dosage registered was 180 ml (Holstebro Hospital) [22] and the lowest was 8 ml (Odense Hospital). The GBCA exposure took place in the period 1998–2008; and exposure was most frequent during 2001–2006 (89.3%). Forty patients (61.5%) had only been exposed to one type of GBCA: 35 to Omniscan®, two to Magnevist®, two to Gadovist® and one to Multihance®. The remaining patients had been exposed to more than one type of GBCAs (n = 15, 23%) or they had been exposed to an unspecified GBCA (n = 10, 15.5%). Figure 2 shows the annual purchase of GBCAs for all Danish radiological departments in the period 1998–2011 [23].

## Discussion

### NSF prevalence

This first nationwide NSF investigation showed a prevalence of 65 per 5.6 million inhabitants, or 12 per million in Denmark. [24]. To our knowledge, Denmark has the highest prevalence of NSF worldwide. It should be noted that the total number of suspected NSF patients reported in Denmark is 99 [25]; this number included deceased patients in whom evaluation of their medical record raised suspicion of NSF, but in whom no clinical or histological examinations were performed. Likewise, a layman could communicate a suspicion of NSF even though the suspicion was not clinically confirmed by a medical doctor. Even in a small country like Denmark, the NSF patients are unevenly distributed, and 75% of the identified NSF patients were exposed to GBCAs at one of two hospitals (Herlev and Skejby Hospital). Initially, back in 2006, the relatively high number of NSF patients observed at Herlev Hospital was thought to be due to institutional hand-on procedures during administration of GBCAs. This assumption was later disproved because similar doses had been used in most Danish radiological departments. The two reports from Skejby and Herlev Hospital differed in terms of prevalence from 4.7% in Skejby [26] to 18% in Herlev [27]. This prominent difference in prevalence is likely explained by differences in MRI inclusion criteria and chronic kidney disease (CKD) stages between the two hospitals. For example, at Herlev Hospital, a group of 102 patients with CKD stage 5 was studied and 18 NSF patients were identified. No NSF patients were identified in patients categorized in CKD1-CKD4. At Skejby Hospital, 17 NSF patients were identified among all 362 GBCA-exposed patients with renal impairment with a mean creatinine clearance of 19 ml/min (SD±15, range 2–88). Besides, Herlev Hospital started their search for NSF patients in 2005, while Skejby Hospital started three years later. A larger number of unrecognized NSF patients may therefore have died at Skejby than at Herlev before being examined.

**Figure 3:**
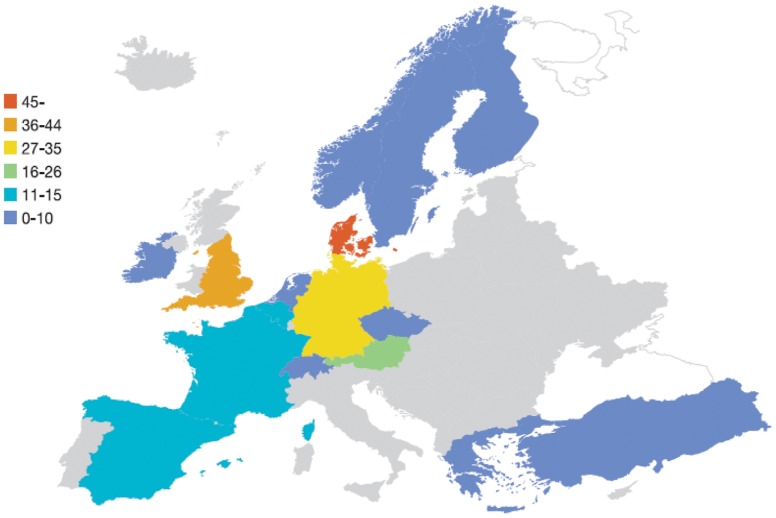
Number of nephrogenic systemic fibrosis patients in Europe, showing the uneven prevalence among countries.

The distribution of NSF is also uneven among countries (Figure 3). While the USA has the highest total number of NSF findings [28]–[32], their prevalence is less than half that found in Denmark.

Bernstein et al listed 20 studies which reported a prevalence of NSF of 0–29.6% [33]. In a retrospective study from Sweden, no NSF patients were found when the NSF diagnosis was searched for among 272 patients with renal insufficiency exposed to GBCA [34]. However, no clinical examinations were performed either. The patients received Omniscan® with a mean dose 0.14 mmol/kg. This dose was equivalent to 17–23 ml Omniscan® per MRI for an average person, while the Danish NSF patients received an average of 31.5 ml of GBCA. Another study from the Auckland region, New Zealand, identified three cases of biopsy-proven NSF and another two cases of clinical NSF within a population of 1.3 million [35]. The dose of GBCA had not been consistently recorded. Hope et al [36] retrospectively reported an NSF prevalence of 1% and stated that it was likely underreported because all patients had not been individually examined and because biopsies were not available for the majority of the patients. These studies support the view that NSF reports cover a wide investigatory spectrum from passive search without any direct patient contact to more active studies involving clinical examination. The study performed by Todd et al [37] in 216 haemodialysis patients was a good example of a pro-active study. They observed cutaneous changes of NSF in 16 (30%) of 54 patients exposed to GBCA but did not obtain skin biopsies which would have completed the study. The nationwide investigation increased the clinical awareness of NSF in Denmark and e.g. 244 patients with renal impairment exposed to GBCA were referred to the dermatological department in Aarhus during 2008–2010 [38]. So by doing, a systematic search and clinical examination ensured diagnosis of 30 NSF patients within a group of 244 patients (ratio 1∶8).

### The challenges in making a nationwide investigation

The present nationwide investigation was challenged by several factors. For example, some potential NSF patients were already deceased from the beginning of the investigation and could obviously not be examined. Other groups of possible NSF patients not included in the nationwide investigation were those who received GBCAs for radiographic purposes [39] or patients who did not have a kidney diagnosis at the time of GBCA exposure, but where the kidney function was already impaired (e.g. diabetic or elderly people). Furthermore, previous experiences showed that chart registrations (coding, medication, GBCA name/use, etc) were inadequate in around 10%. Also, the retrospective design made it impossible to collect all desirable information. Lastly, the NSF findings from individual nephrological departments were challenged by possible differences in procedure and efforts to detect NSF cases at the individual sites.

### Perspectives

The rise in the number of reported NSF cases and subsequent pressure from the media made a nationwide investigation mandatory in Denmark. NSF patients have received medical help and financial compensation for some of their sufferings by means of the Danish health service. Three new cases of NSF have been found in Denmark after the nationwide investigation stopped. Yet, the clinical awareness and active search for NSF cases remain relevant and meaningful; especially in countries in which no or very few NSF patients have yet been identified and where GBCAs have been or are still being used in ways similar to those seen in countries with many NSF cases.

## Conclusion

The outcome of the nationwide investigation revealed 65 NSF patients in a population of 5.6 million inhabitants in Denmark, or 12 per million. This high prevalence of NSF worldwide may be rooted in a high level of awareness of NSF both among Danish doctors, politicians and, not least, the media, combined with the fact that Denmark initiated the first nationwide NSF investigation.

Provenance and peer review: Not commissioned; externally peer reviewed.
