# Correction: Nephrogenic Systemic Fibrosis in Denmark– A Nationwide Investigation

**DOI:** 10.1371/journal.pone.0100407

**Published:** 2014-06-11

**Authors:** The PLOS ONE Staff

There is an error in the dataset analyzed in this article. Following publication, the authors were informed that the Danish Health and Medicines Authority (formerly Danish Medicines Agency) has changed the side effects report so there is no longer a NSF patient who has developed NSF after only being exposed to Multihance®. The patient never had a confirmed NSF diagnosis, so was included in the report by mistake. This error only slightly affects the numbers for the data analyzed and does not alter the conclusions of the article.
